# Advances in Prosthetic Urology

**DOI:** 10.1155/2012/681918

**Published:** 2012-12-04

**Authors:** Gerard D. Henry, Andrew C. Kramer, Rafael E. Carrion, Brian Christine

**Affiliations:** ^1^Department of Urology, Regional Urology, 255 Bert Kouns, Shreveport, LA 71199, USA; ^2^Department of Urology, University of Maryland School of Medicine, Baltimore, MD 21201, USA; ^3^Department of Urology, USF College of Medicine, South Tampa Center for Advanced Healthcare, Tampa, FL 33606, USA; ^4^Erectile Restoration and Prosthetic Urology, Urology Centers of Alabama, Birmingham, AL 35209, USA

The inflatable penile prosthesis (IPP) has become the gold standard treatment for erectile dysfunction among men refractory to medical therapies. Among the many treatments for erectile dysfunction, implantation of a penile prosthesis has been associated with high patient satisfaction rates and low mechanical failure rates. In this special issue, seven articles are presented, including primary research, reviews, and methodological reports, to highlight outcomes related to patient satisfaction with IPPs, advances in surgical placement techniques, and methods for penile size enhancement concomitant with implant placement. 

The safety and efficacy of the IPP have been well documented, but in spite of this, urologists may be reluctant to offer an IPP to older patients due to various concerns, including impaired dexterity of older patients and their ability to operate an inflatable device. To determine the outcomes of and satisfaction with the multicomponent IPP in the elderly male, Villarreal and Jones retrospectively assessed patients using chart review and telephone interview. To analyze overall patient satisfaction with IPPs with a consistent approach, Bernal and Henry conducted a review of the literature over the past 20 years. Nine articles met inclusion criteria for analysis and data collation. Despite the fact that varied metrics were used to determine patient satisfaction, they found that patients in general were very satisfied with their three-piece IPPs and restoration of sexual function, and they identified common reasons for patient dissatisfaction.

The number one patient complaint after IPP placement is loss of penile length. One IPP company has recently remade a product that has longer length cylinders than those formerly available. However, traditionally, longer cylinders were believed to lack axial rigidity. Henry et al. present a prospective, multicenter research study, performed on the new product to address this concern. In a methodology report, Hakky et al. present an overview of various techniques performed concomitantly with IPP placement surgery to enhance penile length and girth. Outcomes can be improved by combining the use of adjunct surgical techniques; these adjuvant procedures are a key addition in the armamentarium for the serious implant surgeon.

In two methodological articles, surgical techniques and outcomes are described related to surgical treatment of erectile dysfunction. First, Martinez et al. describe common surgical techniques for treatment of Peyronie's disease, a clinical condition that interferes with erectile function. Despite attempts to uncover the pathophysiology behind Peyronie's disease, it remains an enigma, with a reported incidence that is rising, in part because more men come forth to seek treatment. Second, Karpman reports on a streamlined approach for infrapubic placement of an IPP. A better understanding of operative techniques and recent clinical outcome studies have led to an evolution of the original infrapubic approach with significant contributions by Dr. Perito as discussed in the erratica. Small incisions and efficient operative maneuvers can shorten operative times and expedite postoperative recovery.

Finally, Henry et al. present a review of outcomes for subarachnoid versus general anesthesia during IPP surgery. The leading patient complaint during the perioperative period for penile prosthesis implantation is postoperative pain, while emesis and urticaria also affect the procedure's perceived success. This paper retrospectively reviews 90 consecutive, primary inflatable penile prosthetic operations performed by a single surgeon at one private medical center.

As erectile dysfunction is highly prevalent in our society, increasing with age, and life expectancy continues to increase, continued research and development regarding IPPs remain an important area of work.

*Gerard D. Henry* *Andrew C. Kramer* *Rafael E. Carrion* *Brian Christine*

